# Quality Physical Intervention Activity for Persons with Down Syndrome

**DOI:** 10.1100/tsw.2007.20

**Published:** 2007-01-10

**Authors:** Meir Lotan

**Affiliations:** ^1^ Zvi Quittman Residential Centers, Israel Elwyn, Jerusalem, Israel; ^2^ Department of Physical Therapy, Academic College of Judea and Samaria, Ariel, Israel; ^3^ Office of the Medical Director, Division for Mental Retardation, Ministry of Social Affairs, Jerusalem, Israel

**Keywords:** Down syndrome, physical activity, Israel

## Abstract

Persons with Down syndrome (DS) are at risk for a life of inactivity that can result in a multitude of medical problems including heart and vascular diseases. This review presents findings regarding the physical status of individuals with DS, as well as proper interventions found to improve the physical fitness and general health for this population. This review was written with the intent to suggest practical directions in planning and implementing quality physical intervention programs for this population.

### INTRODUCTION

About 1 in 800–1,000 of live-born children, irrespective of gender, ethnic, or racial group[1], have Down syndrome (trisomy 21)[2]. The additional chromosomal material results in an array of potential problems with higher prevalence than in the general population[3]. For example: about 40% of Down syndrome (DS) children will have cardiac abnormalities ranging from the simple arterial duct to the atrioventricular septal defect[4], with a high risk of secondary pulmonary hypertension[5], hypothyroidism[6] as well as obesity[7]. The fact that many individuals with intellectual disability such as DS maintain a sedentary life style, experience poorer health, and have less access to health care than the general population[8], suggests that these individuals are at risk for a multitude of secondary health problems. Yet on the other hand, it was established that appropriate intervention might improve the physical condition of this population[9]. The present article will review the benefits of physical activity in the general population, screen the physical state of individuals with DS, and suggest several intervention programs that have been found useful for this population.

### BENEFITS OF PHYSICAL ACTIVITY

Evidence continues to mount on the benefits of physical activity for the well-being of body, mind, and the reduction of risk from chronic disease[10]. A growing body of research that examines the relationship of physical activity to physiological, functional, and psychological outcomes in many disease situations[10,11,12,13] has found that physical activity is associated with physical and psychological benefits[14]. Several studies[10,15] showed that physical activity improved "psychological well-being" (the way in which we deal with stress and mental functioning, such as decision making, planning, and short-term memory), reduced anxiety, and promoted healthier sleep patterns. Mounting evidence negatively correlates between exercise, anxiety, stress, and depression[16].

Other positive effects of regular physical activity are an improvement in cardiovascular and respiratory muscle function, a reduction in coronary artery disease risk factors (i.e., elevated systolic and diastolic blood pressures, or abnormal serum lipid profiles) and a decreased level of overall mortality and morbidity[17]. The benefits also may include enhanced feelings of well-being, and enhanced performance at work, recreation, and sports activities[17]. Recommendations for daily physical activities were published by the CDC[18]:

- Physical activity need not be strenuous to achieve health benefits.
- Men and women of all ages benefit from a moderate amount of daily physical activity. The same moderate amount of activity can be obtained in longer sessions of moderately intense activities (such as 30 min of brisk walking) as in shorter sessions of more strenuous activities (such as 15–20 min of jogging).
- Additional health benefits can be gained through greater amounts of physical activity. Adults who maintain a regular routine of physical activity that is of longer duration or of greater intensity are likely to derive greater benefit. However, because risk of injury also increases with greater amounts of activity, care should be taken to avoid excessive amounts.
- Previously sedentary people who begin physical activity programs should start with short sessions (5–10 min) of physical activity and gradually build up to the desired level of activity.
- Adults with chronic health problems, such as heart disease, diabetes, or obesity, or who are at high risk for these conditions, should first consult a physician before beginning a new program of physical activity. Men over age 40 and women over age 50 who plan to begin a new program of vigorous activity should consult a physician to be sure they do not have heart disease or other health problems.

Despite those specific recommendations and related knowledge on the subject of physical activity and health, a closer look at the physical state of individuals with intellectual disability, is somewhat alarming.

### PHYSICAL FITNESS OF INDIVIDUALS WITH INTELLECTUAL DISABILITY

In several studies, individuals with intellectual disability (ID) were found to lack in physical fitness when compared to peers without ID[19,20,21,22,23]. Measures of poor fitness performance were demonstrated in cardiovascular endurance[24], body composition[25,26], muscular strength and endurance[27,28], and flexibility[29].

Fernhall et al.[24] went so far as to state that the physical fitness of this population was so poor that cognitively disabled 20- to 30-year-old men present the cardiovascular equivalent of people without cognitive disorders who are 10–20 years older or men suffering from chronic heart failure. Many reasons have been suggested for this population’s low level of fitness:

- Sedentary life style[21,30,31]
- Low motivation for participation in physical activities[32]
- Psychological or physiological barriers, or motor passivity[33]
- Physical characteristics, such as short stature[34]
- Lack of coordination and efficiency[35]
- Lack of motivation during testing and a tendency to stop when uncomfortable[36]

Of all the factors examined, it was found that "inactive life style" was the most harmful to physical fitness[21,31,37]. This trend of individuals with ID to stay less active than their siblings and spend significantly more time indoors, showing a preference for indoor activities, was also found among individuals with DS[38].

Sedentary life style is associated with high risk for developing obesity, coronary arterial diseases, and heart attacks[39]. Moreover, it was established that the lack of physical fitness of individuals with multiple disabilities can lead to early aging phenomena and states of illness compared with peers with no such disorders[40]. The assumption mentioned above was proven by a longitudinal study from Canada. In this study, adults with ID were monitored for their physical fitness and compared with a control group. The findings of this program disclosed that the physical fitness levels of the participants were low when compared to those without a disability and declined over the 13-year period. In addition, the magnitude of change over the 13 years, as compared to those without a disability, was greater for male and female participants for body mass index and percentage of body fat, and for female participants for cardiovascular endurance and sit ups. It appeared that adults with ID such as DS might be particularly at risk for declining health associated with aging and low physical fitness. Due to these alarming data, it is clear that intensive habilitative efforts must be introduced to reduce complications and decrease the consequences accompanying old age in this population[41].

### EXERCISE PROGRAMS FOR PERSONS WITH INTELLECTUAL DISABILITY

Findings showed that regular exercise can improve the functional status and can decrease the level of assistance that people with disabilities may need in order to perform activities of daily living by increasing muscular strength, endurance, flexibility, balance, and cardiovascular and respiratory efficiency[42]. Exercise can also reduce the incidence of secondary health complications in persons with disabilities (loss of cardiorespiratory and muscular function, metabolic alterations, and systemic dysfunctions), which may maintain or enhance quality of life[43].

The positive contributions that exercise programs entail have led to the implementation of various intervention programs for individuals with ID. These programs included:

- Stair climbing[44]
- Walking, running, stretching, and aerobic exercises[31]
- Floor and flexibility exercises[23]
- A mile run, use of a rowing machine, weight lifting, bicycle training[45,46]
- Treadmill training[21,47,48]
- Walking[49]

Individuals participating in these programs have improved in muscular endurance[31], physical fitness and reduction of pulse per minute[45], muscle strength[21,50], functional skills[47], and an improved health perception as deduced from reduced visitations to the infirmary[51]. To support physical therapy (PT) programs and in light of the success of previous exercise programs for individuals with ID, it was deduced that for individuals with DS, physical activity has important implications impacting on health, longevity, and productivity[21,33]. Thus, similar activity programs have been implemented for individuals with DS at all ages.

### INTERVENTION PROGRAM FOR CHILDREN WITH DOWN SYNDROME

All areas of development are equally important. Nevertheless, in the first months of life, physical development remains the underlying foundation for all future progress. Babies learn through interaction with their environment. In order to do so, an infant must have the ability to move freely and purposefully.

The ability to explore one's surroundings, to reach and grasp toys, to turn one's head in order to follow a moving object with one's eyes, to roll over, to crawl in pursuit of a desired objective — all of these behaviors are mainly dependent on gross as well as fine motor development. Such physical, interactive abilities foster understanding and mastery of the environment, and their importance to overall development stresses the implication of early intervention programs.

Furthermore, the general fitness level of children, adolescents, and adults with DS is even lower than other people with ID[52,53], and this is believed to have both a motivational and physiological basis[54,55]. It is suggested that this poor state may be partly due to syndrome-specific conditions, such as heart problems, but it seems also that there is a lack of expectations in programming. Individuals with DS have a tendency to become obese in childhood[56], which leads to an adolescent and adult who is less likely to participate in physical activity. Physical activity programs have challenged such a poor beginning for the young child with DS. It was believed that appropriate professional involvement can advance the child with DS, and that early intervention would prevent secondary complications beyond the primary limitations compelled by DS itself[57]. In light of such beliefs, several intervention programs have been suggested around 1980’s for young children with DS.

Connolly and Russell[58] concluded that an interdisciplinary, early intervention program was needed in order to help the participants in earlier attainment of many developmental tasks and enhanced functioning of the family unit. Based on re-evaluating the results (three long-term follow-ups), it was found that achievements of the early intervention program for children with DS had long-lasting results[59]. Thus, it was concluded that participants in the early intervention program gained a foundation for subsequent learning and development[60,61].

Another example of an attempt to evaluate specific motor training for children with DS was conducted in Sweden[62]. An intervention group consisted of 14 children and a control group of six children. The treatment began when the babies were 3 months old and lasted until they began to walk, focusing on increasing muscle tone, reducing incorrect patterns of movement, training typical movement patterns, and stimulating trunk rotation. The treated children performed better than the control children in four areas measured: gross motor, fine motor, kinesthetic perception, and tactile perception

Children with DS have been found to develop walking later than non-DS children, and even later than children with other developmental disabilities[63]. In order to forward walking age in this population, a special walking program was implemented. Seven infants ranging in age from 8–11 months were practiced daily on a treadmill by their parents. All infants responded by producing alternating steps[64]. In a similar intervention, the experimental group learned to walk with help and to walk independently significantly faster than the control group. The results suggest that treadmill training should be considered as an intervention approach for young children with DS[64]. Furthermore, the results provide evidence that, with training and support, parents/caregivers can use these treadmills in their homes to help their infants with DS learn to walk earlier than they normally would[65].

### INTERVENTION PROGRAM FOR ADULTS WITH DOWN SYNDROME

Heller et al.[66] found that a group of adults with DS, who participated in a fitness and health education program for 12 weeks (three practice days per week), changed their attitudes towards exercise, showed more positive expected outcomes and fewer cognitive-emotional barriers, and improved life satisfaction. These findings were very promising since they imply that an appropriate educational-physical program will enhance the participation of individuals with DS in such programs.

Two groups of researchers have performed a jog/walk program[67,68]. Despite the fact that the earlier group could only show minute improvements in walking ability in a group of 14 adolescents with DS after a 10-week jog/walk program, the later group[68] concluded that adults with DS were able to improve their aerobic capacity when performing a systematic and well-designed aerobic training program.

Two treadmill training programs that lasted between 12 weeks and 6 months were held for young (mean age = 24.5 years) and aged adults (mean age = 63 years) with DS[69,70]. Both programs showed significant improvement in muscle strength and dynamic balance, supporting a positive effect. Carmeli et al.[71] implemented a pain-free treadmill walking program for adults with DS and arterial occlusive disease. Some of the participants showed significant improvements in walking speed, distance, and duration. Pain levels were reduced in individuals suffering from intermittent claudication. Blood hemodynamic parameters also showed significant improvements. In conclusion, this program demonstrated that beside improvements of the functional capacities, medical benefits can also be achieved by a low-intensity treadmill walking program for adults with DS. An aerobic 16-week rowing ergometry training regimen in young adults with DS improved exercise endurance and work capacity of participants[72].

The accumulation of evidence suggests that various physical activity programs for individuals with DS can positively and significantly improve numerous health characteristics associated with primary and secondary elements of this group of clients. It is also evident that such programs can be implemented with different age groups and result with appropriate improvements for the trainee. Thus, it is recommended to implement such programs throughout the life span of individuals with DS. In order for the reader to gain some practical information for implementing such programs, the next part of this article will convey some clinical suggestions.

### CLINICAL IMPLICATIONS

This part of the article will reintroduce the reader to basic elements of an exercise program, characteristics of individuals with DS that might interfere with implementing such a program, and suggestions for general exercise guidelines. To prescribe a beneficial exercise program properly, one must have a basic understanding of the elements of exercise physiology and energy metabolism, as well as of the cardiovascular, respiratory, and musculoskeletal responses to exercise and training. Such specific knowledge is mandatory when constructing an appropriate activity program for any population[73]. At the end of the article, an activity program implemented in Israel will be presented. The basic elements all sport activities should entail are cardiovascular exercise, strength training, balance, and flexibility[74,75]. When taking into account the specific characteristics of individuals with DS, the following elements should be included.

#### Cardiovascular Exercise

Due to low levels of cardiovascular fitness of individuals with DS, this is the most important element to consider when initiating an activity program. Recommended modalities include walking, jogging, stationary cycling, and low-impact aerobic dance[36]. The recommended protocol for the general population is training at an intensity of 60–80% of an individual's maximal heart rate (MHR), 3–5 days a week, for 20–60 min per session. But for individuals with DS who usually show very low levels of aerobic fitness, reaching primary levels of 30–50% of the calculated MHR and gradually raising the intensity of the program is recommended. MHR can be obtained from a graded exercise test or can be roughly predicted by subtracting the age of the individual from 220. A suggested starting protocol is a 5- to 10-min continuous activity, 1–2 days a week. Once the participant achieves this level, the intensity can be increased to 40–60% MHR, for 10–30 min, 3–4 days a week. In the later stages of the training program, intensity should be increased to 60–75%, for 15–60 min, 3–5 days a week[36]. Individuals can progress and regress through stages. A training effect is typically seen 16–35 weeks into a program[76], but for untrained individuals, such as many with DS, results might be measurable even after 5–8 weeks of training[45,48].

It is important to monitor heart rate constantly in order to determine the intensity level of the activity and to avoid early-onset fatigue. Recording these data as well as time and distance parameters can be also used as a measurement for the participant's achievements. Some simple test fields for measuring the aerobic level of participants are the Kooper test, also known as the 12-min walk test[77,78], as well as the Energy Expenditure Index (EEI), derived from the following formula[79,80]:

$$\frac{\text{Mean heart rate during exercise} - \text{Mean heart rate during rest}}{\text{Velocity (in meters per second)}}$$

### Strength Training

Due to poor levels of muscular strength found in individuals with DS by several researchers[28,81,82], strength training should be a primary focus in training programs. The goal should be to maximize strength in the large muscle groups. Training intensity should be 70–80% MHR for three sets of 8–12 repetitions. A training effect is typically seen 10–12 weeks into the program. Circuit training is appropriate for individuals with DS. A sample program can include 2-min stations with 30- to 60-sec rest intervals between each station.

Program efficiency should be monitored by collecting data, such as number of lifts (of a constant weight), maximal available load for a specific muscle or muscle groups (only after a proper warm up or at the end of each session), duration of the sessions, resting heart rate, or pulse per minute during training. Body composition measurements are also a good way to follow an individual's strength training progress. To compute body composition for adults with DS, special regression equations have been constructed % Fat = 13.545 + 0.487 (waist circumference, cm) – 0.52 (forearm circumference, cm) – 0.155 (height, cm) + 0.077 (weight, kg)[83].

### Balance Training

Suomi and Koceja[84] found that individuals with ID possess inferior dynamic and static equilibrium abilities compared with the non-ID population. As a group, individuals with DS have been found on numerous occasions[85,86] to score significantly lower than non-DS individuals with ID in the area of balance. The gross motor scores of this population were also significantly lower for a group with DS than for children without DS[86].

Program efficiency should be monitored by using pre to post measurements for balance and equilibrium performance. Tests could be improvised (walking a straight line, walking a high beam, standing/jumping on one leg, etc.) or might use standardized measurements, such as Modified Bass Test, Balance Beam Speed Test 1 (forward walking), and Balance Beam Speed Test 2 (sideward walking)[87], or elements of the Papcsy-DePaepe test and the Bruininks test for measuring balance as they where validated for this population[88].

### Flexibility

Flexibility is a common area of exercise training for the general population. However, because of the hypermobility and joint laxity that is common in DS, it is not a recommended activity for this population.

## BARRIERS TO PARTICIPATION IN EXERCISE PROGRAMS

When aiming at successful participation of individuals with DS in a physical activity program, some limitations should be acknowledged:

- **Limitations set by the physical environment:** Ruuskanen and Parkatti[89] noted that the type of residence influences exercise participation. Adults living in nursing homes were less likely to exercise than the non-nursing home residents.

- **Limitations set by personal characteristics on participation in exercise programs:** Among the general population of older adults, health status is often a predictor of exercise participation. In the study by Ruuskanen and Parkatti[89], poor health status was reported by 60% of the women and 38% of the men as the main reason for not participating in exercise. On the other hand, a study by Turk et al.[90], regarding the health status of 63 women with developmental disability, found a high prevalence of several health conditions that could have an impact on exercise participation including pain (84%), musculoskeletal conditions (59%), and bowel and bladder problems (56%). In spite of these conditions, 83% of the sample reported engaging in at least one common physical activity including swimming, walking, using exercise equipment, and weight lifting. Data from a newer inquiry[91] showed that in adults with cerebral palsy, exercise participation was not related to health status or level of impairment. These studies refute the notion that persons with disabilities are not able to exercise because of physical or cognitive impairments.
- **Limitations set by close human environment:** Several studies have demonstrated the importance of family/caregiver involvement in exercise and weight reduction programs[92,93]. These findings are supported by the findings of Heller et al.[91], suggesting that the most significant determinant of exercise participation of persons with developmental disabilities was their caregiver’s perceived benefits of enrolment in such a program.

### PRECAUTIONS BEFORE PHYSICAL ACTIVITY

- Atlantoaxial instability (AAI) is a severe cervical disorder that occurs in 17% of the population with DS and is characterized by increased laxity between the first and second cervical vertebrae. A possible side effect of this joint laxity is that the vertebrae may sublux and cause spinal cord injury. It is important for an individual with DS to find out if they have AAI before participation in physical activity. Contact sport activities are contraindicated when AAI is present[94,95].
- Congenital heart defects are seen in about 40% of individuals with DS, but are now usually corrected soon after birth. If a congenital heart defect is not corrected right after birth and not healed over time, it will lower the person's life span. Mitral valve prolapse is significantly present in this population, with 44.4–57% occurrence as compared to individuals without DS at 5–10% occurrence[96]. Therefore, all individuals with DS should be evaluated by a physician prior to initiation of a physical activity program, including their cardiac state[97]. Written consent should be given by the physician and the heart rate constantly monitored throughout practice.
- Thyroid hormonal deficiencies are also common in this population and cannot be detected unless appropriate blood testing is done. Hypothyroidism was found in 30% of children with DS in one study[98]. Traditional symptoms of hypothyroidism that might effect participation in an exercise program are fatigue, weakness, weight gain or increased difficulty in losing weight, muscle cramps and frequent muscle aches, depression or irritability[99]. Hypothyroidism can be difficult or even impossible to diagnose on clinical grounds in a person with DS because of major overlap of symptoms with normal features of the syndrome[100]. Hypothyroidism can have a significant effect on participation in any physical activity and, therefore, its presence should be disconfirmed before physical intervention is implemented.
- Abnormal energy expenditure and substrate utilization can also be present in individuals with DS. Therefore, in order to avoid lowering already inadequate intakes of several vitamins and minerals, physical exercise programs for individuals with DS should combine a balanced diet with vitamin and mineral supplementation[101].
- Impaired sympathetic response to exercise has been found in individuals with DS[102]. Such a problem can cause attenuated heart rate response to exercise, which was found predictive of increased mortality and coronary heart disease incidence[103].

- Anemia can be associated with DS and may cause fatigue and reduced ability to participate in physical activity[104]. This condition should be disconfirmed before initiation of a strenuous physical program for this population.

### GENERAL EXERCISE GUIDELINES FOR INDIVIDUALS WITH DOWN SYNDROME

Guidelines for exercise programs for individuals with DS should be[105]:

- Collect pre-exercise medical data on each participant in order to evaluate contraindications and precautions when initiating the program.
- Obtain physician consent.
- Take into consideration the effects of medications on the body in relation to exercise.
- Create a multidimensional activity program that entails endurance, strength, and balance optimal for health and functional benefits[74].
- Make the program fun to participate in.
- If exercise machines are involved, label them with pictures and provide verbal encouragement.
- Provide augmented constant supervision.
- Ensure constant monitoring of heart rate through exercise duration.
- Start the program with light activity that is enjoyable and pain free.
- Provide more visual guidance than verbal instruction[106].
- Incorporate motivational techniques (i.e., token reward system) to improve adherence[36].
- Gradually elevate the program's level of intensity.
- Construct a follow-up program, teaching the clients themselves to record their own information to improve long-term adherence and empowerment.
- Ensure an emergency procedure plan for all programs, especially community-based programs[74].
- Make socially acceptable elements in the program.

Referring to physical activity of individuals with disabilities, Gignac[14] commented that if we substitute relatively meaningless activities for highly valued activities, then it will be effective in maintaining positive physical activity behavior and achieving changes. Fujiura et al.[107] supported this argument and stressed the importance of including physical activity programs for individuals with DS in the community.

The next program is a unique intervention program for individuals with DS that incorporates fun, social interaction, and meaningfulness in accordance with the remarks of Gignac and others[14,21,33,107].

### DANCE AND DRUMS

A physical intervention program that meets a multitude of positive aspects has been running in Israel for about 10 years with a group of seven young individuals with DS aged 22–25 years. Since childhood, this group has participated in movement and music recreation activities. Using the artistic product of this activity, they started to present a show that presented their drumming and dancing skills (see Fig. 1) within their residential center at holidays and special occasions. As time went by, their achievements led to a growing interest in their performance, leading to a slow and steady stream of requests for external performances. In order to respond to the growing demand and upgrade their professional performance, the

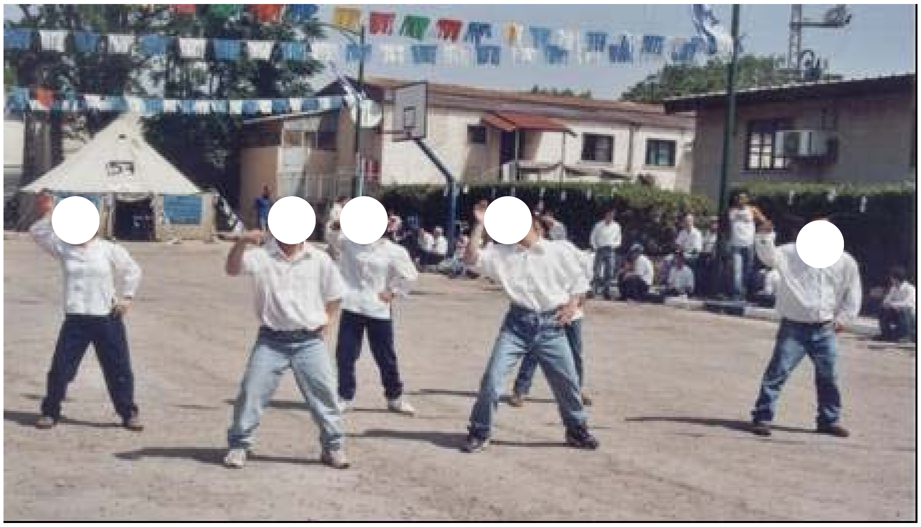

**FIGURE 1.** The dance group during performance.

group now meets for three, 2-h practice sessions a week, with increasing practice intensity before performance dates. In the past 4 years, a group of non-DS/ID children from a regular school have been integrated into the original group of DS dancers.

The benefit of the program lays in the fact that it incorporates intense, high-level physical activity, several times per week, intertwined with the outgoing/happy nature of individuals with DS; it holds a strong motivation appeal (no dropouts over a 10-year period). The success of the program suggests that when the motivational aspects of a program are high enough, even individuals with DS who usually prefer a sedentary life style can maintain a continuous healthy and active life.

The concept and outcome of this program correspond with the conclusions by Stratford and Ching[108] who claim that creative aspects, such as music, movement, and dance, can significantly effect the development of children with DS. The inclusion of a group of non-DS dancers/singers in the group enabled the participants with DS to further expose their friendly, outgoing nature, and spiced up their performance.

### CONCLUSIONS

This article was written with the intent to introduce the poor present physical status of individuals with DS, and to suggest some assistance in planning and implementing quality physical intervention programs for individuals with DS of all ages. Such programs should not be considered as separate aspects, but interwoven throughout the different life stages and activities of the child, adolescent, and adult with DS.

Aspects that were mentioned as the basis of a good physical activity (cardiovascular exercise, strength training, and balance), as well as the guidelines for such a program (mentioned above), should be addressed in the planning stage and kept during the implementation of the physical program. Programs that provide only fitness routines, or games experiences or remedial-type instruction, can be developmentally limiting for the participant with DS. A broad habilitational concept should take into account for the physical development of the individual with DS, but also the person's social, emotional, and cognitive growth.

The author highly recommends the implementation of programs that will incorporate motivational factors, starting at an early stage in life. Such a combination can lead to long-lasting health benefits for this population.

### ACKNOWLEGDMENTS

I wish to thank the participants of the drum and dance group, and the management and staff of "Neve Kinneret" for their collaboration.

## References

1. George, T. and Capone, M.D. (2001) Down syndrome: advance in molecular biology and neurosciences. *J. Dev. Behav. Pradiatr.* **22,** 40–59.
2. Ferencz, C., Rubin, J.D., Loffredo, C.A., and Magee, C.A., Eds. (1993) *Epidemiology of Congenital Heart Disease. The Baltimore-Washington Infant Study 1981–1989.* Futura, Mount Kisco, NY. pp.123–167.
3. Van Vliet, G. (2005) How often should we screen children with Down’s syndrome for hypothyroidism? *Arch. Dis. Child.* **90,** 557–558.
4. Freeman, S.B., Taft, L.F., Dooley, K.J., Allran**,** K., Sherman, S.L., Hassold**,** T.J., Khoury, M.J., and Saker**,** D.M. (1998) Population-based study of congenital heart defects in Down syndrome. *Am. J. Med. Genet.* **80,** 213–217.
5. Luckstead, E.F. (2005) *Cardiovascular Disorders, Essential Adolescent Medicine*. Greydanus, D.E., Patel, D.R., and Pratt H.D., Eds. McGraw-Hill, New York. pp. 125–152.
6. Yang, Q., Rasmussen, S.A., and Friedman, J.M. (2002) Mortality associated with Down’s syndrome in the USA from 1983 to 1997: a population-based study. *Lancet* **359,** 1019–1025.
7. Harris, N., Rosenberg, A., Jangda, S., O'Brien, K., and Gallagher, M.L. (2003) Prevalence of obesity in International Special Olympic athletes as determined by body mass index. *J. Am. Diet Assoc.* **103,** 235–237.
8. Public Health Service (US) (2002) Closing the Gap: A National Blueprint for Improving the Health of Individuals with Mental Retardation. Report of the Surgeon General's Conference on Health Disparities and Mental Retardation. Department of Health and Human Services (US), Washington, D.C. pp. xii.
9. Ordonez, F.J., Rosety, M., and Rosety-Rodriguez, M.(2006) Influence of 12-week exercise training on fat mass percentage in adolescents with Down syndrome. *Med. Sci. Monit.* **12(10),** 416–419.
10. Biddle, S.J.H., Fox, K.R., and Boutcher, S.H. (2000) *Physical Activity and Psychological Well-Being*. Routledge, London.
11. Minor, M.A. and Brown, J.D. (1993) Exercise maintenance of persons with arthritis after participation in a class experience. *Health Educ. Q.* **20,** 83–95.
12. Rall, L.C. and Roubenoff, R. (1996) Body composition, metabolism, and resistance exercise in patients with rheumatoid arthritis. *Arthritis Care Res.* **9,** 151–156.
13. Stenstrom, C.H. (1994) Therapeutic exercise in rheumatoid arthritis. *Arthritis Care Res.* **7,** 190–196.
14. Gignac, M.A.M. (2003) Leisure time physical activity and well-being: learning from people living with arthritis. *J. Rheumatol*. **30(11),** 2299–2301.
15. Hunt, A. (2003) Musculoskeletal fitness: the keystone in overall well-being and injury prevention. *Clin. Orthop.* **(409),** 96–105.
16. North, T.C., McCullagh, P., and Tran, Z.V. (1990) Effect of exercise on depression. *Exerc. Sport Sci. Rev*. **18,** 379–415.
17. Franklin, B.A., Whaley, M.H., and Howley, E.T. (2000) *ACSM's Guidelines for Exercise Testing and Prescription*. 6th ed. Lippincott Williams & Wilkins, Philadelphia.
18. CDC (1999) A Report of the Surgeon General: Physical Activity and Health in Adults. Retrieved December 2006 from http://www.cdc.gov/nccdphp/sgr/adults.htm
19. Pitteti, K.H., Jackson, J.A., Mays, M.S., Fernandez, J.E., and Stubbs, N.B. (1988) Comparison of the physiological profiles of Down and non-Down Syndrome mentally retarded individuals. In *Proceedings of the Annual Conference of the Human Factors Association of Canada*. pp. 45–48.
20. Pitteti, K.H., Jackson, J.A., Stubbs, N.B., Campbell, K.D., and Battar, S.S. (1989) Fitness levels of adult Special Olympics participants. *Adapted Phys. Act. Q.* **6,** 254–270.
21. Pitetti, K.H. and Tan, D.M. (1991). Effects of a minimally supervised exercise program for mentally retarded adults. *Med. Sci. Sports Exerc.* **23(5),** 594–601.
22. Pitetti, K.H. and Boneh, S. (1995) Cardiovascular fitness as related to leg strength in adults with mental retardation. *Med. Sci. Sports Exerc.* **27(3),** 423–428.
23. King, D. and Mace, E. (1990). Acquisition and maintenance of exercise skills under normalized conditions by adults with moderate and severe mental retardation. *Ment. Retard.* **28(5),** 311–317.
24. Fernhall, B., Pitetti, K.H., Rimmer, J.H., McCubbin, J.A., Rintala, P., Miller, A.L., Kittredge, J., and Burkett, L.N.(1996). Cardiorespiratory capacity of individuals with mental retardation including Down syndrome. *Med. Sci. Sports Exerc.* **28(3),** 366–371.
25. Fox, R.A., and Rotatori, A.F. (1982). Prevalence of obesity among mentally retarded adults. *Am. J. Ment. Defic.* **87,** 228–230.
26. Rimmer, J.H., Braddock, D., and Fujiura, C. (1993) Prevalence of obesity in adults with mental retardation: implications for health promotion and disease prevention. *Ment. Retard.* **31,** 105–110.
27. Horvat, M., Pitetti, K.H., and Croce, R. (1997) Isokinetic torque, average power and flexion/extension ratios in nondisabled adults and adults with mental retardation. *J. Orthop. Sports Phys. Ther.* **25,** 395–399.
28. Pitetti, K.H., Climstein, M., Mays, M.J., and Barrett, P.J. (1992) Isokinetic arm and leg strength of adults with Down's syndrome: a comparative study. *Arch. Phys. Med. Rehabil.* **73,** 847–850.
29. Reid, G., Montgomery D.L., and Seidl, C. (1985) Performance of mentally retarded adults on the Canadian Standardized Test of Fitness. *Can. J. Public Health* **76,** 187–190.
30. Pitetti, K.H. and Campbell, K.D. (1991). Mentally retarded individuals--a population at risk? *Med. Sci. Sports Exerc.* **23,** 586–593.
31. Merriman, W.J., Barnett, B.E., and Jarry, E.S. (1996). Improving fitness of dually diagnosed adults. *Percept. Mot. Skills* **83(3 Pt 1),** 999–1004.
32. Halle, J.W., Gabler-Halle, D., and Chung, Y.B. (1999). Effects of a peer mediated aerobic conditioning program on fitness levels of youth with mental retardation: two systematic replication. *Ment. Retard.* **37(6),** 435–448.
33. Fernhall, B. and Tymeson, G.T. (1988).Validation of a cardiovascular fitness field test for adults with mental retardation. *Adapted Phys. Act. Q.* **5,** 49–59.
34. Dobbins, A.D., Garron, R., and Rarick, G.L. (1981). The motor performance of educable mentally retarded and intellectually normal boys after covariate control for differences in body size. *Res. Q. Exerc. Sport* **52,** 1–8.
35. Seidl, C., Montgomery, D., and Reid, G. (1989) Stair stepping efficiency of mentally handicapped and nonmentally handicapped adult females. *Ergonomics* **32,** 519–526.
36. Rimmer, J.H. (1994). *Fitness and Rehabilitation Programs for Special Populations*. William C. Brown, Dubuque, IA.
37. Bickum, D. (1995). *The History of Graded Exercise Testing in Cardiac Rehabilitation*. Microform Publication, University of Oregon.
38. Sharav, T. and Bowman, T. (1992) Dietary practices, physical activity, and body-mass index in a selected population of Down syndrome children and their siblings. *Clin. Pediatr. (Phila.)* **31(6),** 341–344.
39. Draheim, C.C., Williams, D.P., and McCubbin, J.A. (2002) Prevalence of physical inactivity and recommended physical activity in community-based adults with mental retardation. *Ment. Retard.* **40(6),** 436–444.
40. Vaccaro, P. and Mahon, A.D. (1989). The effects of exercise on coronary heart disease risk factors in children. *Sports Med.* **8(3),** 139–153.
41. Ohry, A. (2003) Premature aging: a danger to life expectancy and quality of life of the disabled. *Shikumada* **20,** 65–68. [Hebrew]
42. Rimmer, J. H. (1999) Health promotion for people with disabilities: the emerging paradigm shift from disability prevention to prevention of secondary conditions. *Phys. Ther.* **79(5),** 495–502.
43. Noreau, L. and Shephard, R.J. (1995). Spinal cord injury, exercise and quality of life. *Sports Med.* **20(4),** 226–250.
44. French, R., Silliman, L.M., Ben-Ezra, V., and Landrien-Seiter, M. (1992) Influence of selected reinforcers on the cardiorespiratory exercise behavior of profoundly mentally retarded youth. *Percept. Mot. Skills* **74,** 584–586.
45. Tomporowski, P.D. and Ellis, N.R. (1985) The effects of exercise on the health, intelligence, and adaptive behavior of institutionalized severely and profoundly mentally retarded adults: a systematic replication. *Appl. Res. Ment. Retard.* **6,** 465–473.
46. Tomporowski, P.D. and Ellis, N.R. (1984) Effects of exercise on the physical fitness, intelligence, and adaptive behavior of institutionalized mentally retarded adults. *Appl. Res. Ment. Retard.* **5,** 329–337.
47. Lotan, M., Isakov, E., and Merrick, J. (2004) Improving functional skills and physical fitness in children with Rett syndrome. *J. Intellect. Disabil. Res.* **48(8),** 730–735.
48. Lotan, M., Isakov, E., Kessel, S., and Merrick, J. (2004) Physical fitness and functional ability of children with intellectual disability: effects of a short-term daily treadmill intervention. *TheScientificWorldJOURNAL* **4,** 449–457.
49. Bauer, D. (1981) Aerobic fitness for the severely and profoundly mentally retarded. *Pract. Point.* **5(4),** 1–41.
50. Chanias, A.K., Reid, G., and Hoover, M.L. (1998) Exercise effects on health-related physical fitness of individuals with an intellectual disability: a meta- analysis. *Adapted Phys. Act. Q.* **15,** 119–140.
51. Lotan, M. (2003) Physical Therapy Intervention Programs for Seniors with Intellectual Disabilities. A presentation during a panel on elderly with intellectual disability. The Annual International Conference of the American Association for Mental Retardation (AAMR), Chicago.
52. Burns, Y. and Gunn, P. (1993) *Down Syndrome: Moving Through Life*. Chapman and Hall, London.
53. Block, M.E. (1991) Motor development in children with Down syndrome: a review of the literature. *Adapted Phys. Act. Q.* **8,** 179–209.
54. Fernhall, B., Tymeson, G., Millar, L., and Burkett, L. (1989) Cardiovascular fitness testing and fitness levels of adolescents and adults with mental retardation including Down syndrome. *Educ. Train. Ment. Retard.* **24,** 133–137.
55. Pitetti, K.H., Climstein, M., Campbell, K.D., Barrett, P.J., and Jackson, J.A. (1992) The cardiovascular capacities of adults with Down syndrome: a comparative study. *Med. Sci. Sports Exerc.* **24,** 13–19.
56. Cronk, C.E., Chumlea, C.W., and Roche, A.F. (1985) Assessment of overweight children with trisomy 21. *Am. J. Ment. Defic.* **89,** 433–436.
57. Cicchetti, D. and Beeghly, M. (1990) An organizational approach to the study of Down syndrome: contributions to an integrative theory of development. In *Children with Down Syndrome: A Developmental Perspective*. Cicchetti, D. and Beeghly, M., Eds. Cambridge University Press. pp. 29–62.
58. Connolly, B. and Russell, F. (1976) Interdisciplinary early intervention program. *Phys. Ther.* **56(2),** 155–158.
59. Connolly, B.H., Morgan, S.B., Russell, F.F., and Fulliton, W.L. (1993) A longitudinal study of children with Down syndrome who experienced early intervention programming. *Phys. Ther.* **73(3),** 170–181.
60. Connolly, B., Morgan, S., Russell, F.F., and Richardson, B. (1980) Early intervention with Down syndrome children: follow-up report. *Phys. Ther*. **60(11),** 1405–1408.
61. Connolly, B.H., Morgan, S., and Russell, F.F. (1984) Evaluation of children with Down syndrome who participated in an early intervention program. Second follow-up study. *Phys. Ther*. **64(10),** 1515–1519.
62. Bjornhage, L., Lagerwall, B., Ericsson-Sagsjo, A., and Waldenstrom, E. (1990). Early motor training for children with Down syndrome. In *Looking Up at Down syndrome*. Chigier, E., Ed. Freund Publishing, London. pp. 163–171.
63. Kokubun, M., Haishi, K., Okuzumi, H., and Hosobuchi, T. (1995) Factors affecting age of walking by children with mental retardation. *Percept. Mot. Skills* **80(2),** 547–552.
64. Ulrich, B.D., Ulrich, D.A., Collier, D.H., and Cole, E.L. (1995) Developmental shifts in the ability of infants with Down syndrome to produce treadmill steps. *Phys. Ther*. **75(1),** 14–23.
65. Ulrich, D.A. Ulrich, B.D., Angulo-Kinzler, R.M., and Yun, J. (2001) Treadmill training of infants with Down syndrome: evidence-based developmental outcomes. *Pediatrics* **108(5),** e84.
66. Heller, T., Hsieh, K., and Rimmer, J.H. (2004) Attitudinal and psychosocial outcomes of a fitness and health education program on adults with Down syndrome. *Am. J. Ment. Retard*. **109(2),** 175–185.
67. Millar, A.L., Fernhall, B., and Burkett, L.N. (1993) Effects of aerobic training in adolescents with Down syndrome. *Med. Sci. Sports Exerc*. **25(2),** 270–274.
68. Tsimaras, V., Giagazoglou, P., Fotiadou, E., Christoulas, K., and Angelopoulou, N. (2003) Jog-walk training in cardiorespiratory fitness of adults with Down syndrome. *Percept. Mot. Skills* **96(3 Pt 2),** 1239–1251.
69. Tsimaras, V.K. and Fotiadou, E.G. (2004) Effect of training on the muscle strength and dynamic balance ability of adults with Down syndrome. *J. Strength Cond. Res*. **18(2),** 343–347.
70. Carmeli, E., Kessel, S., Coleman, R., and Ayalon, M. (2002) Effects of a treadmill walking program on muscle strength and balance in elderly people with Down syndrome. *J. Gerontol. A Biol. Sci. Med. Sci.* **57(2),** M106–110.
71. Carmeli, E., Barchad, S., Masharawi, Y., and Coleman, R. (2004) Impact of a walking program in people with Down syndrome. *J. Strength Cond. Res*. **18(1),** 180–184.
72. Varela, A.M., Sardinha, L.B., and Pitetti, K.H. (2001) Effects of an aerobic rowing training regimen in young adults with Down syndrome. *Am. J. Ment. Retard*. **106(2),** 135–144.
73. Ashe, M.C. and Khan, K.M. (2004) Exercise prescription. *J. Am. Acad. Orthop. Surg*. **12(1),** 21–27.
74. Cress, M.E., Buchner, D.M., Prohaska, T., Rimmer, J., Brown, M., Macera, C., Dipietro, L., and Chodzko-Zajko, W. (2005) Best practices for physical activity programs and behavior counseling in older adult populations. *J. Aging Phys. Act*. **13(1),** 61–74.
75. American College of Sports Medicine (2004) Physical activity programs and behavior counseling in older adult populations. *Med. Sci. Sports Exerc.* **36(11),** 1997–2003.
76. Fernhall, B. (1997) Mental retardation. In *ACSM's Exercise Management for Persons with Chronic Diseases and Disabilities*. Durstine, L., and Moore G.E., Eds. American College of Sports Medicine, Human Kinetics Publishers, Portland.
77. Campo, L.A., Chilingaryan, G., Berg, K., Paradis, B., and Mazer, B. (2006) Validity and reliability of the modified shuttle walk test in patients with chronic obstructive pulmonary disease. *Arch. Phys. Med. Rehabil*. **87,** 918–922.
78. Grant, S., Corbett, K., Amjad, A.M., Wilson, J., and Aitchison, T. (1995) A comparison of methods of predicting maximum oxygen uptake. *Br. J. Sports Med.* **29(3),** 147–152.
79. Rose, J. Gamble, J.G. Lee, J., and Haskell, W.L. (1991) The energy expenditure index: a method to quantitate and compare walking energy expenditure for children and adolescents. *J. Pediatr. Orthop*. **11(5),** 571–578.
80. Rose, J., Gamble, J.G., Burgos, A., Medeiros, J., and Haskell, W.L. (1990) Energy expenditure index of walking for normal children and for children with cerebral palsy. *Dev. Med. Child Neurol.* **32(4),** 333–340.
81. Croce, R.V., Pitetti, K.H., Horvat, M., and Miller, J. (1996) Peak torque, average power, and hamstrings/quadriceps ratios in nondisabled adults and adults with mental retardation. *Arch. Phys. Med. Rehabil*. **77(4)**, 369–372.
82. Angelopoulou, N., Tsimaras, V., Christoulas, K., Kokaridas, D., and Mandroukas, K. (1999) Isokinetic knee muscle strength of individuals with mental retardation, a comparative study. *Percept. Mot. Skills* **88(3 Pt 1),** 849–855.
83. Rimmer, J.H., Kelly, L.E., and Rosentswieg, J. (1987) Accuracy of anthropometric equations for estimating body composition of mentally retarded adults. *Am. J. Ment. Defic*. **91(6),** 626–632.
84. Suomi, R. and Koceja, D.M. (1994) Postural sway patterns of normal men and women and men with mental retardation during a two-legged stance test. *Arch. Phys. Med. Rehabil*. **75(2),** 205–209.
85. Kokubun, M., Shinmyo, T., Ogita, M., Morita, K., Furuta, M., Haishi, K., Okuzumi, H., and Koike, T. (1997) Comparison of postural control of children with Down syndrome and those with other forms of mental retardation. *Percept. Mot. Skills* **84(2),** 499–504.
86. Connolly, B.H. and Michael, B.T. (1986) Performance of retarded children, with and without Down syndrome, on the Bruininks Oseretsky Test of Motor Proficiency. *Phys. Ther.* **66(3),** 344–348.
87. Tsigilis, N., Zachopoulou, E., and Mavridis, T. (2001) Evaluation of the specificity of selected dynamic balance tests. *Percept/ Mot/ Skills* **92(3 Pt 1),** 827–833.
88. DePaepe, J.L. and Ciccaglione, S. (1993) A dynamic balance measure for persons with severe and profound mental retardation. *Percept. Mot. Skills* **76(2),** 619–627.
89. Ruuskanen, J.M. and Parkatti, T. (1994). Physical activity and related factors among nursing home residents. *J. Am.*

*Geriatr. Soc.* **42(9),** 987–991.
90. Turk, M.A., Overeynder, J.C., and Janicki, M.P., Eds. (1995) *Uncertain Future - Aging and Cerebral Palsy: Clinical Concerns.* New York State Developmental Disabilities Planning Council, Albany.
91. Heller, T., Ying, G.S., Rimmer, J.H., and Marks, B.A. (2002) Determinants of exercise in adults with cerebral palsy. *Public Health Nurs.* **19(3),** 223–231.
92. Fox, R.A., Rosenberg, R., and Rotatori, A.F. (1985). Parent involvement in a treatment program for obese retarded adults. *J. Behav. Ther. Exp. Psychiatry* **16(1),** 45–48.
93. Thorbecke, P.J. and Jackson, H.J. (1982) Reducing chronic drooling in a retarded female using a multi-treatment package. *J. Behav. Ther. Exp. Psychiatry* **13(1),** 89–93.
94. Pueschel, S.M. (1998) Should children with Down syndrome be screened for atlantoaxial instability? *Arch. Pediatr. Adolesc. Med.* **152(2),** 123–125.
95. Cremers, M.J., Bol, E., de Roos, F., and van Gijn, J. (1993) Risk of sports activities in children with Down's syndrome and atlantoaxial instability. *Lancet* **342(8870),** 511–514.
96. Pueschel, S.M. and Werner, J.C. (1994) Mitral valve prolapse in persons with Down syndrome. *Res. Dev. Disabil.* **15(2),** 91–97.
97. Pastore, E., Marino, B., Calzolari, A., Digilio, M.C., Giannotti, A., and Turchetta, A. (2000) Clinical and cardiorespiratory assessment in children with Down syndrome without congenital heart disease. *Arch. Pediatr. Adolesc. Med.* **154(4),** 408–410.
98. Tuysuz, B. and Beker, D.B. (2001) Thyroid dysfunction in children with Down's syndrome. *Acta Paediatr.* **90(12),** 1389–1393.
99. Canaris, G.J., Steiner, J.F., and Ridgway, E.C. (1997) Do traditional symptoms of hypothyroidism correlate with biochemical disease? *J. Gen. Intern. Med.* **12(9),** 544–550.
100. Prasher, V.P. (1995) Prevalence of psychiatric disorders in adults with Down syndrome. *Eur. J. Psychiatry* **9,** 77–82.
101. Luke, A., Sutton, M., Schoeller, D.A., and Roizen, N.J. (1996) Nutrient intake and obesity in prepubescent children with Down syndrome. *J. Am. Diet Assoc.* **96(12),** 1262–1267.
102. Eberhard, Y., Eterradossi, J., and Therminarias, A. (1991) Biochemical changes and catecholamine responses in Down's syndrome adolescents in relation to incremental maximal exercise. *Ment. Defic. Res.* **35(Pt 2),** 140–146.
103. Lauer, M.S., Okin, P.M., Larson, M.G., Evans, J.C., and Levy, D. (1996) Impaired heart rate response to graded exercise. Prognostic implications of chronotropic incompetence in the Framingham Heart Study. *Circulation* **93(8),** 1520–1526.
104. Pitetti, K.H., Climstein, M., Campbell, K.D., Barrett, P.J., and Jackson, J.A. (1992) The cardiovascular capacities of adults with Down syndrome: a comparative study. *Med. Sci. Sports Exerc.* **24,** 13–19.
105. Mahler, D.A., Froelicher, V.F., Miller, N.H., et al (1995) *ACSM's Guidelines for Exercise Testing and Prescription.* 5th ed. Williams & Wilkins, Baltimore, MD.
106. Le Clair, D.A. and Elliott, D. (1995) Movement preparation and the costs and benefits associated with advance information for adults with Down syndrome. *Adapted Phys. Act. Q.* **12(3),** 239–249.
107. Fujiura, G.T., Fitzsimons, N., Marks, B., and Chicoine, B. (1997) Predictors of BMI among adults with Down syndrome: the social context of health promotion. *Res. Dev. Disabil.* **18(4),** 261–274.
108. Stratford, B. and Ching, E.Y. (1989) Responses to music and movement in the development of children with Down's syndrome. *J. Ment. Defic. Res.* **33(Pt 1),** 13–24.
